# Plant-Based Dietary Fibers and Polysaccharides as Modulators of Gut Microbiota in Intestinal and Lung Inflammation: Current State and Challenges

**DOI:** 10.3390/nu15153321

**Published:** 2023-07-26

**Authors:** Yu Shen, Mingming Song, Shihao Wu, Hongbo Zhao, Yu Zhang

**Affiliations:** 1 Heilongjiang Provincial Key Laboratory of New Drug Development and Pharmacotoxicological Evaluation, College of Pharmacy, Jiamusi University, Jiamusi 154007, China; shenyu0406@126.com (Y.S.);; 2 College of Rehabilitation Medicine, Jiamusi University, Jiamusi 154007, China

**Keywords:** dietary fibers, plant polysaccharide, prebiotics, lung inflammation, intestinal inflammation, gut–lung axis, phytotherapy

## Abstract

Recent research has underscored the significant role of gut microbiota in managing various diseases, including intestinal and lung inflammation. It is now well established that diet plays a crucial role in shaping the composition of the microbiota, leading to changes in metabolite production. Consequently, dietary interventions have emerged as promising preventive and therapeutic approaches for managing these diseases. Plant-based dietary fibers, particularly polysaccharides and oligosaccharides, have attracted attention as potential therapeutic agents for modulating gut microbiota and alleviating intestinal and lung inflammation. This comprehensive review aims to provide an in-depth overview of the current state of research in this field, emphasizing the challenges and limitations associated with the use of plant-based dietary fibers and polysaccharides in managing intestinal and lung inflammation. By shedding light on existing issues and limitations, this review seeks to stimulate further research and development in this promising area of therapeutic intervention.

## 1. Introduction

Chronic inflammatory diseases affecting mucosal sites, such as the intestine and lungs/airways, are becoming increasingly prevalent worldwide [1,2]. These diseases encompass inflammatory bowel disease (IBD), including ulcerative colitis (UC) and Crohn’s disease, as well as lung conditions such as asthma, bronchiectasis, chronic obstructive pulmonary disease (COPD), and cystic fibrosis (CF). COPD, which includes chronic bronchitis and emphysema, has been responsible for 3.23 million deaths in 2019 and emerged as the third leading cause of death globally [3]. Similarly, IBD has witnessed a global increase in prevalence over time and has emerged as a significant health concern [4].

Recent studies focusing on gut and lung inflammatory diseases have revealed a close relationship between the gut and lung microbiota, which is referred to as the gut–lung axis (GLA) [5]. The GLA operates in a bidirectional manner, where lung inflammatory diseases impact the gastrointestinal system, and vice versa [5]. For instance, long-term smoking not only degrades lung tissues and causes inflammation but also leads to the infiltration of circulating immune cells in the gut, resulting in gut dysbiosis and an increased risk of intestinal diseases such as IBD [6]. Likewise, a recent cross-sectional cohort study called Respiratory Health in Northern Europe (RHINE), conducted among participants from North European countries, found an increased risk of asthma in IBD patients, particularly among women [7]. Moreover, it is important to note that genetic susceptibility to inflammatory bowel disease (IBD) can also increase the risk of interstitial lung disease, a condition characterized by inflammation and fibrosis [8,9]. Several factors have been proposed to explain the link between gut and lung diseases, including their shared embryological origin and the influence of gut microbiota and their metabolites on the regulation of lung immunity and inflammatory responses [7]. As a result, the lung and gut exhibit an intricate interconnection, and both should be carefully considered in individuals affected by either disease.

The current management of IBD involves the use of various medications, including corticosteroids, aminosalicylates, immunosuppressive agents, antibiotics, oral small molecules, and biologics such as anti-tumor necrosis factor TNF-α [10]. However, these medications come with a range of side effects, ranging from mild symptoms, like headaches, nausea, and abdominal pains, to more severe complications, such as infertility, photosensitization, opportunistic infections, diabetes mellitus, hemolytic anemia, hypertension, granulocytosis, and osteoporosis [10]. Similar observations have been made in the case of COPD and asthma, where many potential drugs have failed to demonstrate efficacy in clinical trials or have limited application due to side effects such as headaches, nausea, diarrhea, and dose-limiting effects [11].

Due to the intricate connection between the gut and the lungs, and the undesirable side effects of current medications used for gut and lung inflammatory diseases, researchers have shifted their focus towards exploring traditional and alternative medicinal approaches, particularly those based on plant dietary fibers (DFs) and plant polysaccharides. These plant-derived compounds have demonstrated promising potential for preventing and alleviating gut and lung inflammation through their ability to modulate the microbiota. Notably, plant-based polysaccharides exhibit diverse biological functions, including antioxidative, anti-inflammatory, immunomodulatory, antitumor, hypoglycemic, and microbiota modulation capabilities [12]. Such findings hold significant promise for the development of novel therapeutic interventions to address gut–lung-axis diseases.

DFs consist of polymers of monosaccharides that are indigestible and not absorbed in the gastrointestinal tract due to the lack of proper hydrolyzing enzymes. They are primarily found in plants (grains, fruits, and vegetables), fungi, and algae [13,14,15]. Dietary fibers can be categorized into non-starch polysaccharides (NPSs), such as cellulose, hemicellulose, pectin, beta-glucans, resistant starches, and lignin [15]. Additionally, low-molecular-weight non-digestible oligosaccharides such as fructooligosaccharides (FOSs), galactooligosaccharides (GOSs), xylooligosaccharides (XOSs), and inulin are sometimes included in the definition of dietary fibers [15]. Non-carbohydrate components like lignin, cutin, saponin, and suberin can also function as dietary fibers [14].

**Figure 1:**
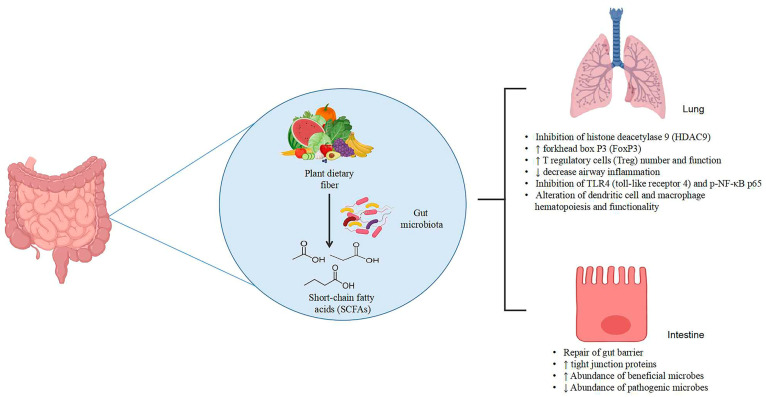
Mechanism of plant dietary fibers and polysaccharides against gut and lung inflammation. Abbreviations: ↑, increased; ↓, decreased.

This review critically discusses the utilization of plant-based polysaccharides and dietary fibers for ameliorating gut and lung inflammatory diseases, primarily through microbiota modulation. Table 1 provides a summary of alterations in microbial composition associated with intestinal and lung inflammatory diseases within the gut–lung axis. Table 2 presents recent studies on the effects of plant dietary fibers and polysaccharides on intestinal inflammatory diseases through gut microbiota modulation. Furthermore, Table 3 lists examples of plant dietary fibers and polysaccharides that have demonstrated the amelioration of lung inflammation via gut microbiota modulation. Figure 1 provides a summarized mechanism of action for plant-based dietary fibers and polysaccharides against gut and lung inflammatory diseases.

## 2. Plant Dietary Fibers and Polysaccharides against Gut and Lung Inflammation

### 2.1. Different Plant Dietary Fibers and Polysaccharides Shape Gut Microbiota and Affect SCFA Production Differently

Despite the availability of various drugs that offer relief for lung inflammation and intestinal inflammatory diseases, tackling gut dysbiosis, a common feature of many gut and lung diseases, as discussed above, remains a significant challenge for these medications. However, dietary fibers (DFs) and polysaccharides have emerged as potential candidates for effectively modulating the gut microbiota and playing a vital role in the prevention and treatment of these conditions. Their ability to target and positively influence the gut microbiota holds promising implications for addressing gut–lung axis diseases more effectively.

A study by Jang et al. [52] demonstrated that different types of DF have differential effects on gut microbial composition and short-chain fatty acid (SCFA) production. In the study, emphysema mice were fed either non-fermentable cellulose or fermentable pectin-rich diets. Both cellulose and pectin lowered pro-inflammatory mediators, including IFN-γ, IL-1β, IL-6, IL-8, IL-18, TNF-α, and TGF-β. However, pectin supplementation (fermentable fiber) exhibited higher anti-inflammatory action than non-fermentable-fiber (cellulose) supplementation in emphysema mice. Regarding gut microbiota composition, at the phylum level, *Bacteroidetes* were the most abundant in the high-pectin diet group. At the family level, *Lactobacillaceae* and *Defluviitaleaceae* were the lowest in the high-pectin diet group compared with the high-cellulose diet group or control.

In addition to the type of dietary fiber (DF), the structural modifications of DF also play a crucial role in its ability to shape the gut microbiota and its preventive effects on lung and gut inflammation. For instance, pectin, a plant-derived water-soluble DF primarily composed of linear chains of galacturonic acid that can be esterified, has been studied in relation to its impact on ulcerative colitis. Research by Fan et al. [75] demonstrated that pectin with a degree of esterification below 50% exhibited a higher preventive potential against ulcerative colitis than pectin with a degree of esterification above 50%. In another study conducted by the same research group, they explored the effects of pectins with different degrees of esterification and dosages on gut microbiota and serum metabolites in a colitis mouse model. These findings highlight the significance of considering structural variations in DF to harness its full potential for modulating gut microbiota and preventing inflammation-related diseases.

The composition changes of major genera, including *Lactobacillus, Bifidobacterium, Akkermansia, Prevotella, Ruminococcus*, and *Oscillospira*, vary widely depending on the degree of esterification and pectin dosage. Another comparative study examined three polysaccharide conjugates derived from dried fresh tea leaves (FTPS), green tea (GTPS), and black tea (BTPS) [29]. These conjugates were tested for their capacity to regulate intestinal homeostasis in DSS-induced colitis mice. While all tea polysaccharide conjugates increased the abundance of *Bacteroides* and *Muribaculaceae* and reduced *Helicobacter* and *Enterococcus* abundance, only FTPS and BTPS exhibited inhibitory effects on *Mucispirillum*. Moreover, the production of SCFAs differed among the three conjugates, with FTPS showing the highest SCFA production, followed by BTPS and GTPS. Overall, FTPS demonstrated better efficacy than BTPS and GTPS in preventing colitis.

Cystic fibrosis (CF) is associated with gut dysbiosis and intestinal inflammation. In a pilot study, Wang et al. [76] investigated whether CF patients’ depleted microbiota had the potential to utilize a prebiotic substrate, such as high-amylose maize starch (HAMS), for increased SCFA production. Various techniques, including metagenomic sequencing, in vitro fermentation, amplicon sequencing, and metabolomics, were employed to examine the HAMS fermentation capacity of the gut microbiome in adults with CF and controls. CF patients exhibited low abundances of taxa associated with HAMS fermentation (*Faecalibacterium, Roseburia*, and *Coprococcus*) and lower levels of acetate production compared to controls. However, there was no difference in the production of butyrate and propionate between CF patients and controls. The study reported that high butyrate production in healthy controls was associated with a high relative abundance of the commensal genus *Faecalibacterium*. Interestingly, in the absence of butyrate-producing *Faecalibacterium* in CF patients, another bacterium, *Clostridium* ss1, competitively fermented HAMS and biosynthesized butyrate. This finding suggests that despite alterations in gut microbiota, the prebiotic effect of DF can be mediated by different taxa, which is in contrast to the notion that the presence of commensal bacteria is necessary to produce the prebiotic effect of DF.

### 2.2. Plant Dietary Fibers and Polysaccharides Alter the Intestinal Barrier in Both Gut Inflammation and Lung Inflammation

The intestinal barrier serves as an important defense mechanism that maintains a healthy balance of inflammatory factors to prevent the entry of pathogens into the body. It consists of four main barrier systems: mechanical, chemical, immune, and biological barriers [77]. Gut dysbiosis, among other factors, can disrupt the integrity of the intestinal barrier by affecting tight junctions in epithelial cells. This disruption can allow the translocation of gut microbes to other body sites, such as the lungs, and promote lung inflammation [54,78].

Plant polysaccharides have been shown to improve lung inflammation by repairing the intestinal barrier. Zhu et al. [58] extracted *Houttuynia cordata* polysaccharide (HCP), which mainly consists of glucose, galactose, arabinose, and rhamnose in a ratio of 3.40:2.14:1.17:1. The inclusion of HCP in the diet of influenza A virus-infected mice not only repaired the intestinal barrier by increasing secretory immunoglobulin A (sIgA) and the tight junction protein zonula occludens-1 (ZO-1) but also reduced proinflammatory cytokines and chemokines such as TNF-α, IL-6, and IFN-α. Furthermore, the anti-inflammatory properties of HCP in the lung and gut were mediated through the inhibition of toll-like receptor 4 (TLR4) and phosphorylated NF-κB p65 in the lung [58]. However, this study did not explore the effect of HCP on gut microbiota and its role in lung inflammation.

In another study, it was observed that the oral administration of high-amylose cornstarch (HCP) to mice infected with influenza A virus led to improvements in intestinal barrier integrity and alleviated gut inflammation. This effect was attributed to the increased levels of intestinal tight junction proteins and secretory IgA (sIgA) in mice. These findings suggest that HCP may have potential therapeutic benefits in managing gut inflammation and enhancing intestinal barrier function during viral infections. Moreover, HCP corrected the alterations in gut microbiota by reducing the relative abundances of pathogenic bacteria Vibrio and Bacillus [57].

The protective effects of plant polysaccharides on the intestinal barrier have also been demonstrated for polysaccharides extracted from sources other than *Houttuynia cordata*. These sources include Chinese yam [26], *Rhinacanthus nasutus* and okara [35], *Scutellaria baicalensis* Georgi [40], Polygonatum sibiricum [34], Cyclocarya paliurus [24], *Rehmannia glutinosa* [33], *Rosa roxburghii* Tratt [22,79], *Dendrobium fimbriatum* Hook [31], and tea [29] in animal models of ulcerative colitis and inflammatory bowel disease (IBD).

### 2.3. Plant-Dietary-Fiber and Polysaccharide Supplementation in Gut and Lung Inflammation in Human Adults (Clinical Studies) and Adult Mice (Preclinical Studies)

Park et al. [80] in a prospective human cohort study (*n* = 219,123 men and 168,999 women, aged 50–71 years, 9-year follow-up) evaluated the effect of dietary-fiber intake on health outcomes. The study reported that the consumption of dietary fiber was associated with a reduced risk of mortality from respiratory diseases in both men and women. In a three-way, cross-over, double-blind, randomized controlled trial, adults with stable asthma were administered a soluble fiber, inulin (12 g per day), for 7 days, leading to improved asthma control and reduced airway inflammation. Additionally, inulin supplementation was found to modulate the gut microbiome, as evidenced by an increase in the relative abundance and absolute numbers of bifidobacteria [56]. These findings suggest that dietary fiber, particularly inulin, may play a beneficial role in improving respiratory health and gut microbiota composition.

In another randomized, double-blind, placebo-controlled, crossover study, soluble-fiber supplementation in healthy people did not only reduce the levels of pro-inflammatory factors such as TNF-α, IL-6, and IL-8 but also improved gut microbiota composition and increased the production of butyrate [81]. The effect of a soluble-fiber meal (containing 3.5 g of inulin and probiotics) on airway inflammation and free fatty acid receptor activity in adults with stable asthma was examined in a pilot study [61]. Soluble-fiber supplementation alleviated airway inflammation, as shown by reductions in total cell count, neutrophils, macrophages, lymphocytes, and IL-8. Further, the observed anti-inflammatory effect of the soluble-fiber meal was found to improve lung function via the upregulation of free fatty acid receptors, GPR41 and GPR43 gene expression.

In a preclinical study conducted on adult mouse models with allergic airway disease (AAD), a pectin-rich diet was found to alleviate allergic airway inflammation. The pectin-rich diet led to alterations in gut microbiota homeostasis, increasing the abundance of the *Bacteroidaceae* and *Bifidobacteriaceae* families. These bacteria have the capacity to ferment soluble fiber into short-chain fatty acids (SCFAs) such as acetate and propionate. The protective effects of the pectin-rich diet were primarily mediated by SCFAs, which promoted dendritic-cell hematopoiesis and functionality. This, in turn, resulted in reduced Th2-cell response, attenuation of allergic inflammation, and improved lung function [68].

Similarly, SCFAs fermented from an inulin-rich diet have been shown to protect against influenza-induced pathology in adult mice. This protection was achieved by altering macrophage hematopoiesis and functionality and preventing the entry of neutrophils into the airways, thereby ameliorating tissue destruction [82]. These findings indicate that dietary fibers, such as pectin and inulin, can modulate gut microbiota and SCFA production, leading to beneficial effects on respiratory health and inflammation.

Among various plant DFs and polysaccharides, *Houttuynia cordata*-extracted polysaccharide has been frequently used as a gut microbiota modulator to alleviate anti-lung inflammatory agents in lipopolysaccharide-induced acute lung injury [63] and influenza virus infection-induced lung injury [44,46,54,57,58]. Other than *Houttuynia cordata*, polysaccharides derived from *Lycium barbarum* [55] and *Ephedra sinica* [49] can also ameliorate lung inflammation in mice by modulating gut microbiota composition. Polysaccharides extracted from *Platycodon grandiflorus* [48] and *Tetrastigma hemsleyanum* [50] have also been reported to possess lung anti-inflammatory response. *Tetrastigma hemsleyanum* polysaccharides could correct antibiotic-induced intestinal mucosal barrier dysfunction and gut inflammation in mice, thus showing an important role in the management of the gut–lung axis [83].

### 2.4. Plant-Dietary-Fiber and Polysaccharide Supplementation in Maternal Rodent Models

Dietary fibers (DFs) or polysaccharides have also been investigated for their potential in preventing asthma in offspring by feeding asthmatic mother mice a DF-rich diet during pregnancy and/or lactation. Thorburn et al. [67] fed a high-fiber diet to female mice with house dust mite (HDM)-induced asthma and observed decreased susceptibility to allergic airway disease (AAD) in their offspring. This effect was mediated indirectly through alterations in the gut microbiota, specifically an increase in *Bacteroidetes* and a decrease in *Firmicutes*, as well as directly through the production of short-chain fatty acid (SCFA) metabolites. The intestinal microbiota fermented the high-fiber diet into SCFAs, which inhibited histone deacetylase 9 (HDAC9) and led to epigenetic modifications of the forkhead box P3 (Foxp3) promoter, resulting in increased Foxp3 expression. Foxp3, in turn, led to an increase in the pool and function of T regulatory cells (Tregs), ultimately ameliorating airway inflammation.

In two separate studies conducted by Hogenkamp et al., mice were supplemented with different mixtures of non-digestible oligosaccharides. One study involved a diet supplemented with short-chain galactooligosaccharides (scGOSs) and long-chain fructooligosaccharides (lcFOSs) in a ratio of 9:1, while the other study used a diet supplemented with scGOSs, lcFOSs, and pectin-derived acidic oligosaccharides (pAOSs) in a ratio of 9:1:2 [66,84]. Both studies reported a reduction in allergic asthma in male offspring. However, no significant differences were found in the production of cytokines such as IL-13, IL-4, IL-5, IL-10, IL-17, IFN-γ, and TNF-α between the control group and the non-digestible-oligosaccharide diet group in either study. Additionally, neither study explored whether the therapeutic effect of the oligosaccharide-rich diet on offspring was mediated by the gut microbiota.

However, a recent study by Yuan et al. [42] demonstrated that supplementation with inulin, a soluble dietary fiber, significantly altered the composition of maternal gut microbiota by increasing SCFA-producing *Bifidobacterium*. This supplementation also attenuated the asthmatic inflammatory response in the offspring. The study employed drinking water containing 10% inulin for supplementation.

### 2.5. Sex Differences in Lung Anti-Inflammatory Effect of Plant DFs and Polysaccharides

Many lung inflammatory diseases, such as asthma, COPD, CF, and acute pneumonia, exhibit sexual dimorphism, with differences in disease susceptibility, severity, and prognosis between males and females [85]. Additionally, sex differences can also impact the microbial populations present in various body parts, including the gut microbiome and lung microbiome, and this is referred to as microgenderome [85]. Therefore, it is possible that dietary interventions involving dietary fiber (DF) against lung inflammation may also show sex-based differences in effectiveness. This hypothesis was recently tested by Tashiro et al. [53] using an ozone-induced airway hyperresponsiveness (AHR) mouse model, where cellulose and pectin were included in the diet. The study found that only the cellulose-rich diet reduced ozone-induced AHR in males, but increased AHR, neutrophilic airway inflammation, and airway injury in females. The sex difference in the efficacy of DF against pulmonary response was attributed to differences in diet-related changes in gut microbiota composition between male and female mice.

Sex-specific effects of DF have also been observed in mouse models of gut inflammation. Isomaltodextrins (IMDs) are starch-based soluble dietary fibers prepared using α-glucosyltransferase and α-amylase enzymes [86]. An interleukin (IL)-10-deficient colitis mouse model was treated with IMDs to examine their effect on colitis and gut microbiota and to assess whether the impact of IMDs was sex-specific [86]. IMD supplementation in female mice reduced alpha diversity and *Coprococcus* abundance, while in male mice, it increased alpha diversity, community richness, and evenness and showed a lesser reduction in *Coprococcus* abundance. These studies highlight the sex-dependent response to DF supplementation of gut microbiota in both gut and pulmonary inflammation, indicating that the therapeutic application of plant DF requires careful adaptation for males and females.

### 2.6. Combination of Plant Polysaccharides with Other Phytochemicals

There have been only a few studies that have explored the therapeutic effects of plant dietary fibers or plant polysaccharides in combination with other phytochemicals. In an interesting study by Ling et al. [44], the efficacy of polysaccharides and flavonoids isolated from *Houttuynia cordata* was tested either alone or in combination against H1N1-induced pneumonia in mice. The combined administration of polysaccharides (80 mg/kg) and flavonoids (100 mg/kg) demonstrated excellent ability to regulate pulmonary homeostasis compared with either therapy alone.

Budesonide is a corticosteroid drug commonly used for airway and gut inflammation [87]. However, long-term use of this drug can lead to several side effects, such as tuberculosis infection, hypersensitivity reactions, and an increased risk of infections. To enhance the efficacy of budesonide and reduce its side effects by lowering the required dose, Verheijden et al. [60] investigated the use of galactooligosaccharides (GOSs) as a dietary adjunct therapy against pulmonary inflammation in a murine model of house dust mite-induced allergic asthma. The combination of budesonide and GOSs exhibited a more potent anti-allergic inflammation effect than budesonide or GOSs alone. However, one limitation of the study was that the authors did not assess the impact of the various treatments on gut microbiota composition.

### 2.7. Mechanisms of Polysaccharides and Dietary Fibers in Reducing Intestinal and Lung Inflammation

The underlying mechanisms responsible for the anti-inflammatory effects of plant DFs and polysaccharides on the lungs remain poorly understood. However, among various mechanisms proposed for the lung and intestine anti-inflammatory properties of plant polysaccharides, mostly are mediated via either changes in gut microbiota composition or SCFAs.

T helper type 17 (Th17) and regulatory T (Treg) cells are important subsets of effector cells derived from CD4 T cells, having an important function in maintaining immune homeostasis in the body. Th17 cells are pro-inflammatory in nature, produce pro-inflammatory molecules, such as IL-17, IL-17A, IL-17F, IL-21, and IL-22, and recruit neutrophils; on the other hand, Treg cells help suppress inflammatory and immune responses by producing anti-inflammatory cytokine IL-10 and transforming growth factor (TGF)-β1 [88,89]. Therefore, Th17-/Treg-cell balance is an important factor in determining inflammatory homeostasis in the body, and gut dysbiosis can affect the Th17-/Treg-cell balance in intestinal and lung inflammatory diseases, such as in IBD and asthma [90,91]. Plant-based DFs and polysaccharides can regulate this Th17-/Treg-cell imbalance and improve gut and lung inflammation by modulating gut microbiota and consequently SCFA production [46,54,67]. For example, *Houttuynia cordata* polysaccharide treatment corrected Th17-/Treg-cell imbalance both in gut-associated lymphoid tissue (GALT) and lungs by increasing the total number of Treg cells, reducing the expression of chemokine CCL20 in the lung, and promoting the migration of Treg cells in Peyer’s patches–mesenteric lymph nodes–lung axis in H1N1-infected mice [54]. SCFAs, particularly acetate, have been reported to be involved in correcting the balance between Th17 and Treg cells in the gut–lung axis [46]. SCFAs can maintain the Th17-/Treg-cell balance via the following mechanism: SCFAs increase the expression of forkhead box P3 (Foxp3) transcription factor via epigenetic modifications of the Foxp3 promoter by inhibiting the histone deacetylase 9 (HDAC9) enzyme; the increased expression of Foxp3, in turn, leads to an increase in the pool and function of T regulatory cells (Tregs), ultimately ameliorating airway inflammation [67].

However, some studies present an alternative view, suggesting that the alleviation of asthmatic symptoms with high-fiber diets, such as those rich in cellulose, may not be always mediated through increased production of intestinal SCFAs but also via the regulation of intestinal microflora composition, which can affect the body’s lipid metabolism [45]. Similarly, Lai et al. [92] showed that oral administration of commensal bacteria *Parabacteroides goldsteinii* MTS01 could significantly inhibit a cigarette-smoke-induced COPD murine model by correcting disturbed amino acid metabolism, reducing intestinal inflammation, improving ribosomal and mitochondrial functions in the intestines, and ameliorating lung inflammation. Therefore, diets rich in plant polysaccharides that can increase the relative abundance of such beneficial bacteria have the potential to ameliorate lung inflammatory diseases.

Interestingly, other studies highlighted that plant DFs’ and polysaccharides’ lung and intestinal anti-inflammatory mechanisms were not dependent on either gut microbiota or microbial fermentation of DFs into SCFAs. For example, Sonoyama et al. [72] reported a reduction in eosinophilic infiltration in the lungs and a reduction in the levels of IL-4 and IL-5 mRNA in ovalbumin-sensitized rats supplemented with raffinose (RAF) and α-linked galactooligosaccharide (GOS) diets. These effects were retained even after cecectomy and antibiotic treatment, which led researchers to suggest that the therapeutic effects of RAF and GOS were not due to their fermentation by bacteria. Similarly, for fructan DFs with β2→1 linkage, such as inulin and fructooligosaccharides, it was observed that their immunomodulatory effect was mainly dependent on their chemical structure, and this effect was mediated not by the gut microbiota but via increases in the number of type 1 T helper (Th1) cells in Peyer’s patches and in the number of dendritic cells and Tregs in mesenteric lymph nodes in mice [93].

As we already discuss above, the gut barrier is an important defense system that prevents the entry of pathogens into the body. The lack of plant DF in diet can increase the permeability of the intestinal barrier to pathogenic microbes and bacterial lipopolysaccharides (LPSs), which, by binding to TLR4, lead to the activation of NF-κB signaling and inflammatory cytokine production [94,95]. Plant DFs and polysaccharides could suppress the expression of TLR4 and p-NF-κB-p65 in the lungs and ultimately inhibit lung inflammation [43,58]. Although bacterial LPSs generally act as activators of TLR4 and play a role in downstream inflammatory activation, in an interesting study, the LPS derived from an intestinal commensal bacterium, *Parabacteroides goldsteinii*, was found to be the main component that ameliorated a cigarette-smoke-induced COPD murine model by acting as an antagonist for TLR4 and consequently reducing the over-expression of proinflammatory cytokines such as IL-1β and TNF-α in the lungs and colon in COPD mice [92].

## 3. Issues and Challenges

The gut–lung axis represents a bidirectional communication pathway between the respiratory and gastrointestinal tracts. Despite the bidirectional nature of this axis, current research primarily focuses on the impact of gut microbiota on lung diseases, with limited studies investigating the influence of lung microbiota on inflammation and whether dietary interventions using plant-based dietary fibers (DFs) and polysaccharides can affect lung-microbiome diversity. This exploration is necessary, because while the gut and lung microbial populations are similar at the phylum level, they differ significantly at the species level [96].

Another challenge lies in accurately defining plant DFs and polysaccharides. While many studies have demonstrated the therapeutic effects of carbohydrates such as inulin, fructooligosaccharides, and galactooligosaccharides in gut and lung inflammation (refer to Table 2 and Table 3), it has been suggested that these low-molecular-weight (LMW) non-digestible carbohydrates should not be considered DFs. Their rapid fermentation in the proximal colon can cause side effects, including flatulence, abdominal distention, and abdominal pain [15]. Therefore, interventions involving polysaccharides should be preferred over oligosaccharide supplementation to maximize the benefits for lung and gut inflammation.

Despite the similarity at the phylum level, the species composition of gut microbiota varies widely among individuals of different races and ethnicities [97]. Generally, studies investigating the therapeutic potential of plant DF and polysaccharide supplementation in gut and lung inflammation have not taken individual variations in microbiota into account, nor have they included an individual’s response to such supplementation. It is crucial to understand these variations, because DF supplementation in individuals with gut dysbiosis may inadvertently worsen the dysbiosis. Therefore, prebiotic recommendations cannot be generalized and require proper safety evaluations of plant-based DFs and polysaccharides in populations with gut and lung inflammation.

Compared with research on probiotics and short-chain fatty acids, research exploring the use of plant polysaccharide-based prebiotics against gut and lung inflammation is limited, especially in clinical studies. High-quality, large-scale, well-designed randomized trials are necessary before plant DFs and polysaccharides can be recommended as gut modulator therapies for individuals with gut and lung inflammatory diseases.

## 4. Conclusions

Plant dietary fibers (DFs) and polysaccharides have shown promising results in preventing gut and lung inflammation by influencing gut microbiota, as supported by both clinical and preclinical studies. This highlights the potential of DFs, including plant polysaccharides, as a prebiotic dietary approach to managing gut and lung inflammation. However, it is essential to address the current research focus, which has been primarily centered on the gut microbiota in the gut–lung axis, with limited attention given to the effects of DFs and plant polysaccharides on the lung microbiome. This knowledge gap is further exacerbated by the predominant emphasis on the bacterial components of the microbiota, while the fungal and viral microbiota of the gastrointestinal tract have been less explored. This limitation hinders a comprehensive understanding of gut and lung inflammation.

Furthermore, there is a scarcity of research on the use of plant polysaccharides, as many studies have predominantly focused on low-molecular-weight (LMW) carbohydrates such as inulin, fructooligosaccharides, and galactooligosaccharides. Therefore, substantial efforts are required to fully explore and harness the prebiotic potential of plant polysaccharides. Addressing these research gaps could not only enhance our understanding of the gut–lung axis but also open new avenues for the development of effective therapeutic interventions using plant-based dietary fibers and polysaccharides to manage gut and lung inflammation.

In conclusion, the modulation of gut microbiota with the administration of plant DFs and polysaccharides shows promise in preventing gut and lung inflammation. However, more research is needed to better understand the effects of DFs and plant polysaccharides on the lung microbiome and to expand our knowledge beyond bacterial microbiota. Additionally, the exploration of plant polysaccharides as prebiotics requires more attention to fully unlock their potential. Advancing our understanding in these areas could facilitate the development of effective dietary strategies for managing gut and lung inflammation.

## Figures and Tables

**Table 1:** Alteration in microbial composition associated with intestinal and lung inflammatory diseases in gut–lung axis.

	Gut Microbiota	Lung Microbiota	
IBD	*↓ Bifidobacterium longum, ↓ Eubacterium rectale, ↓ Faecalibacterium prausnitzii, ↓ Roseburia intestinalis, ↑ Bacteroides, ↑ Ruminococcus torques, ↑ Ruminococcus, ↓ Christensenellaceae, ↓ Coriobacteriaceae, ↓ Clostridium leptum, ↑ Actinomyces* spp.*, ↑ Veillonella* spp.*, ↑ Escherichia coli, ↓ Eubacterium rectum, ↓ Akkermansia muciniphila*	—	[16]
COPD	*Streptococcus parasanguinis*_*B* and *Streptococcus salivarius, Streptococcus vestibularis, Streptococcus sp000187445, Lachnospiraceae*	*↓ Veillonella*, ↑ *Actinomyces*, ↑ *Actinobacillus, ↑ Megasphaera, ↑ Selenomonas, ↑ Corynebacterium, ↑ Streptococcus pneumoniae, ↑ Gemella morbillorum, ↑ Prevotella histicola, ↑ Streptococcus gordonii*	[17,18]
Asthma	↑ *Haemophilus*, ↑ *Streptococcus*, ↑ *Moraxella*, ↑ *Lactobacillus*	↑ *Haemophilus* spp., ↑ *Moraxella catarrhalis, ↑ Streptococcus* spp., *Tropheryma*	[19]
Cystic fibrosis	↑ *Escherichia*, ↑ *Shigella*, ↑ *Enterobacter*, ↑ *Clostridium*, ↑ *Veillonella, ↑ Enterococcus, ↑ Staphylococcus, ↓ Lachnospiraceae, ↓ Ruminococcu, ↓ Roseburia, ↓ Faecalibacterium, ↓ Eubacterium, ↓ Prevotella, ↓ Eggerthella, ↓ Alistipes*	*Pseudomonas aeruginosa, Staphylococcus aureus, H. influenzae, Burkholderia cepacia, Actinobacteria, Proteobacteria*	[19,20]

Abbreviations: ↑, increased in abundance; ↓, decreased in abundance; COPD, chronic obstructive pulmonary disease; IBD, inflammatory bowel disease.

**Table 2:** Effect of plant polysaccharides on intestinal inflammatory diseases via gut microbiota modulation.

Natural Source	Model	Effects on Inflammation Related Biomarkers	Intestinal Microbiota Modulation	References
Buckwheat	TNBS-induced colitis	↓ IL-6, IL-1β, and TNF-α	↑ F/B ratio, ↑ *Oscillospiraceae*, *↑ Oscillibacter*, ↑ SCFAs	[21]
*Rosa roxburghii* Tratt polysaccharide	HFD-induced colitis	↓ TNF-α, IL-6 and IL-1β, ↑ tight junction proteins	↓ *Desulfovibrionaceae*, ↓ *Enterobacteriaceae*, ↑ *Muribaculaceae*, ↑ *Bacteroidaceae*	[22]
*Rubus chingii* Hu unripe fruit polysaccharide	HFD-induced colitis	↓ IL-6, ↓ IL-1β, ↓ TNF-α	*↑* gut microbial diversity, ↓ *Erysipelatoclostridium*, *↓ Negativibacillus*	[23]
*Cyclocarya paliurus* polysaccharide	DSS-induced colitis	↑ IL-10, ↓ IL-1β, ↓ TNF-α	↓ *Akkermansia*, ↓ *Sutterella*, ↓ *AF12*, ↓ *Clostridiaceae_Clostridium*, ↓ *Helicobacter*, ↓ *Prevotella*, ↑ *Lactobacillus*, ↑ *Coprococcus*	[24]
*Smilax china* L. polysaccharide	DSS-induced colitis	↓ TNF-α, ↓ IL-6, ↓ IL-1β, ↑ IL-10	↑ *Lachnospiraceae*, ↑ *Muribaculaceae*, ↑ *Blautia*, ↑ *Mucispirillum*, ↓ *Akkermansiaceae*, ↓ *Deferribacteraceae*, ↓ *Oscillibacter*	[25]
Chinese yam polysaccharide	LPS-stimulated co-culture of Caco-2/Raw264.7 cells	↓ NO, ↓ IL-1β, ↓ TNF-α	↑ *Bifidobacterium*, ↑ *Megasphaera.*	[26]
Fuzhuan brick tea polysaccharide	DSS-induced colitis	↓ IL-6, ↓ IL-1β, ↓ IFN-γ, ↓ TNF-α, ↑ tight junction proteins (Occludin, Claudin-1, and ZO-1), ↑ intestinal barrier function	↑ *Bacteroides*, ↑ *Parasutterella*, ↑ *Collinsella*	[27]
Fuc-S (a sulfated α-L-Fucooligosaccharide)	DSS-induced colitis	↓ TNF-α, ↓ IL-1β, ↓ IL-6, ↓ IL-17A	↓ F/B ratio, ↑ *Akkermansia*, ↑ *Prevotellaceae_UCG_001*, *↓ Eubacterium xylanophilum*, ↓ *Intestinimonas*, ↓ *Ruminococcaceae* UCG-014, ↓ *Oscillibacter*	[28]
Polysaccharide conjugates derived from dried fresh tea leaves, green tea, and black tea	DSS-induced colitis	↓ IL-6, ↓ IFN-γ, ↓ IL-1β, ↓ TNF-α, ↑ IL-10	↑ *Bacteroides*, ↑ *Muribaculaceae*, ↓ *Helicobacter*, ↓ *Enterococcus*	[29]
Pectin with various esterification degrees	DSS-induced colitis	—	*↑ Lactobacillus*, *↑ Bifidobacterium*	[30]
*Rosa roxburghii* Tratt	HFD-induced intestinal barrier dysfunction and inflammation	↓ TNF-α, ↓IL-6, ↓IL-1β; ↑ tight junction proteins (ZO-1, claudin-1, and occludin); ↓ intestinal permeability; ↓ colonic oxidative stress	↓ F/B ratio, ↑ Ruminococcaceae, ↑ Muribaculaceae, ↑ Akkermansiaceae	[22]
*Dendrobium fimbriatum* Hook	DSS-induced colitis	↓ IL-1β, ↓ IL-6, ↓ IL-17A, ↓ IL-17F, ↓ IL-21, ↓ IL-23, ↑ IL-5, ↑ IL-10, ↑ IL-22, ↑ IFN-γ, ↑ TNF-α, ↑ TGFβ	↑ *Romboutsia*, ↑ *Lactobacillus*, ↑ *Odoribacter*, ↓ *Parasutterella*, ↓ *Burkholderia-Caballeronia-Paraburkholderia*, ↓ *Acinetobacter*	[31]
Pectin	LPS-induced inflammation in piglets	↓ TNF-α, ↑ IL-10	↓ *Helicobacter*, ↑ *Olsenella*, ↑ *Bacteroides*, ↑ *Proteus*, ↑ *Eubacterium*	[32]
*Rehmannia glutinosa* polysaccharide	DSS-induced colitis	↑tight junction proteins, ↓ IL-10, ↓ IL-6, ↓ TNF-α, ↑ IL-10	↓ *Bacteroidaceae*, ↑ *Lactobacillus*, ↑ *Alistipes*, ↑ *Lachnospiraceae_NK4A13*	[33]
*Polygonatum sibiricum* polysaccharide	Aged mouse model	↓ IL-23, ↓ IL-6, ↓ IL-1β, ↓ TNFα, ↓ IL-17, ↓ IL-12, ↓ IL-6, ↑ IL4, ↑ IL-10	↑ *Bifidobacterium*, ↑ *Lactobacillus*, ↓ *Escherichia coli*	[34]
*Rhinacanthus nasutus* and okra	AA-induced colitis	↓ IL1β, ↓ IL-2, ↓ IL-6, ↑ IL-10	↑ *Muribaculaceae*, ↓ *Bacteroidaceae*, ↓ *Tannerellaceae*	[35]
*Houttuynia cordata* polysaccharides	DSS-induced colitis	↓ TNF-α, ↓ IL-1β, ↓ IL-6	↑ *Firmicutes, ↑ Bacteroides*, ↓ *Proteobacteria*	[36]
*Lonicera japonica Thunb*	DSS-induced colitis	↑ IL-2, ↑ TNF-α, ↑ IFN-γ	↑ *Bifidobacterium, ↑ Lactobacilli, ↓ Escherichia coli, ↓ Enterococcus*	[37]
*Crataegus pinnatifida*	DSS-induced colitis	↓ IL-1β, ↓ IL-6, ↓ TNF-α	↑ *Alistipes*, ↑ *Odoribacter*	[38]
*Morinda citrifolia* L.	DSS-induced colitis	↓ TNF-α, ↓ IL-17	↑ *Dubosiella*, ↑ *Muribaculaceae*, ↑ *Ruminococcaceae_UGG-014*, ↑ *Ruminococcus_1*, ↓ *Bilophila*, ↓ *Campylobacter*, ↓ *Escherichia-Shigella*, ↓ *Ochrobactrum*, ↓ *Vibrio*	[39]
*Scutellaria baicalensis* Georgi.	DSS-induced colitis	↓IL-6, ↓IL-1β, ↓TNF-α	↑ *Bifidobacterium*, ↑ *Firmicutes*, ↑ *Lactobacillus*, ↑ *Roseburia*	[40]

Abbreviations: ↑, increased; ↓, decreased; AA, acetic acid; DSS, dextran sulfate sodium; F/B ratio, Firmicutes/Bacteroidetes ratio; HFD, high-fat diet; IL, interleukin; IFN, interferon; LPS, lipopolysaccharide; TGFβ, transforming growth factor β; TNBS, 2,4,6-trinitrobenzene sulfonic acid; TNF, tumor necrosis factor; ZO-1, zonula occludens-1 (also known as Tight junction protein 1).

**Table 3:** Effect of plant polysaccharides on lung inflammatory diseases mediated via gut microbiota modulation.

Polysaccharide Intervention	Disease	Anti-Inflammatory Outcomes in Lungs	Effects on Intestinal Microflora	Reference
Pear extract	Preclinical asthma mouse model and randomized, double-blind clinical studies	↓ pro-inflammatory cytokines, including IgE, IL-4, IL-5, and IL-13	↑ Bifidobacterium and Eubacterium	[41]
Inulin (10%)	OVA- and Al (OH)3-induced asthma in SD rats	Attenuation of the asthmatic inflammatory response in the offspring	↑ SCFA-producing bacteria (mainly Bifidobacterium) in maternal intestinal microflora; alteration in intestinal microflora composition of offspring	[42]
*Astragalus membranaceus* polysaccharide (25, 50, and 100 mg/kg)	Bleomycin-induced pulmonary fibrosis	↓ damage and collagen deposition in lung tissue; ↓ inflammatory cytokines TNF-α, IL-6, and IL-1β levels; ↓ apoptosis	Restoration of gut microbiota homeostasis; ↑ *Lactobacillus* and *Akkermansia*; ↓ *Lachnoclostridium*, *Clostridium*, and *Erysipelatoclostridium*	[43]
*Houttuynia cordata* polysaccharides (80 mg/kg) and flavonoids (100 mg/kg) either alone or in combination	Influenza virus H1N1-infected mice	Combined therapy showed more potent effect than monotherapy; inhibition of inflammatory-cell infiltration and production of chemokines or pro-inflammatory cytokines such as MCP-1, IL-8, TNF-α, IL-6, and IL-1 β;	Restoration of microflora composition, ↑ *Bacteroidetes*-to-*Firmicutes* (B/F) ratio	[44]
Cellulose-rich diet (30%)	OVA- and Al(OH)_3_-induced asthma in C57BL/6J mice	↓ inflammatory cell infiltration around the bronchus and blood vessels; normalization of airway epithelial structure; ↓IL-4; ↓IgE	↑*Peptostreptococcaceae*	[45]
*Houttuynia cordata* polysaccharides (40 mg/kg)	H1N1-induced pneumonia in antibiotic-treated BALB/c mice (termed BALB/c-ABX mice)	Significant amelioration of inflammation in lungs of BALB/c mice	Attenuation of pathological change in intestine; ↓ *Bacteroidetes* at phylum level; ↓ *Bacteroides* and ↑ *f_Lachnoospiraceae* at genus level, ↑ gut microbial diversity; ↑ acetate	[46]
*Astragalus* polysaccharides	Lipopolysaccharide-induced inflammatory lung injury	Alleviation of histopathological abnormalities in lung tissues; ↓ neutrophils infiltration; inhibition of LPS-induced lung inflammation	Change in colonic microbiota composition; ↑ short-chain fatty acid (SCFA)-producing genera such as *Oscillospira*, *Akkermansia*, and *Coprococcus*	[47]
*Platycodon grandiflorus* polysaccharide (75, 150, and 300 mg/kg) and platycodin D alone and together	Chronic bronchitis in SD rats induced via smoking	Improvement in histopathological abnormalities; ↓ excess mucus secretion; improved immunological imbalance in lungs of CB model rat	—	[48]
*Ephedra sinica* polysaccharide	PM- and OVA-induced asthma in mice	↓ eosinophils in BALF; ↓ serum Ig-E, IL-6, TNF-α, and IL-1β; ↓ airway inflammation	↑ *Bacteroides*, *Lactobacillus*, *Prevotella*, *Butyricicoccus*, and *Paraprevotella*; ↓ *Enterococcus* and *Ruminococcus*; ↑ acetic acid, propionic acid, butyric acid, isobutyric acid, valeric acid, isovaleric acid, and isohexanic acid	[49]
Polysaccharides from Tetrastigma hemsleyanum Diels et Gilg	LPS-induced ARDS in Balb/c mice	↓ IL-6, ↓ TNF-α, inhibition of pulmonary inflammation via TLR2/TLR4 pathway	—	[50]
Polysaccharide-rich ethanol precipitate fraction of black tea	Particulate matter (PM)-induced lung injury in BALB/c mice	↓ oxidative stress and inflammation in the lungs; ↓ IL-6, CXCL1, CXCL15, and MDA	↑ Lachnospiraceae, ↓ Lactobacillaceae	[51]
High-cellulose (20%) or high-pectin diet (20)	Cigarette-smoke (CS)-exposed C57BL/6 mouse emphysema model	↓ Alveolar destruction and inflammation in BALF; ↓ macrophages and neutrophils in BALF; ↓ mRNA expression of IFN-γ, IL-1β, IL-6, IL-8, IL-18, TNF-α, TGF-β	↑ SCFAs, bile acids, sphingolipids; ↑ Bacteroidetes; ↓ *Lactobacillaceae*; ↓ *Defluviitaleaceae*; ↓ *Oscillospiraceae*	[52]
Cellulose- or pectin-enriched diet	Ozone-induced airway hyperresponsiveness in C57BL/6 mice	↓ ozone-induced AHR, neutrophilic airway inflammation, and airway injury in female but not male mice; cellulose-based diets ameliorated ozone-induced airway hyperresponsiveness in male but not female mice	↓ Firmicutes, ↑ Proteobacteria and Verrucomicrobia in pectin-fed mice; ↑ *Proteus* and *Lactobacillus*, and ↓ *Parabacteroides*, *Clostridiales*, and *Lachnospiraceae* in pectin- versus cellulose-fed mice	[53]
*Houttuynia cordata* polysaccharides	H1N1-induced acute lung injury in C57BL/6 mice	Restoration of Th17/Treg cells balance in the lung, ↓ CCL20 expression	Restoration of Th17/Treg-cell balance of gut mucosa-associated lymphoid tissue (GALT)	[54]
*Lycium barbarum* polysaccharide	Asthma (OVA-induced mouse model)	↓ lung injury; ↓ TNF, IL-4, IL-6, MCP-1, and IL-17A in plasma and BALF	↑ *Lactobacillus* and *Bifidobacterium*; ↓ *Firmicutes*, *Actinobacteria*, *Alistipes*, and *Clostridiales*	[55]
Soluble fiber (inulin 12 g/day), soluble fiber + probiotic (inulin 12 g/day + multi-strain probiotic >25 billion CFU)	Randomized, double-blind, three-way cross-over trial involving asthmatic patients	No differences between groups in asthma control or airway inflammation	No differences between groups in SCFA levels	[56]
*Houttuynia cordata* polysaccharide (40 mg/kg/day)	H1N1 virus-infected mice	↓ lung inflammation via inhibition of TLR signaling pathway, and IL-1β production and promotion of IL-10 production	↓ intestinal barrier damage; ↑ ZO-1 expression; ↓ relative abundance of pathogenic bacterial genera *Vibrio* and *Bacillus *	[57]
*Houttuynia cordata* polysaccharide (20 mg/kg; 40 mg/kg)	Influenza A virus (IAV) H1N1-mediated pneumonia in BALB/c mice	Amelioration of pulmonary injury; inhibition of TNF-α, IL-6, IFN-α, RANTES, MCP-1, MIP-1α; and IP-10 production	↓ intestinal goblet cells; ↑ intestinal physical and immune barrier; ↑ tight junction protein (ZO-1) in intestine	[58]
Pectin (30%)	Asthma (ozone-exposed mice)	↓ ozone-induced airway hyperresponsiveness	↑ serum short-chain fatty acids	[59]
GOS (1 or 2.5 *w/w*%) alone or with budesonide	HDM-induced asthma in BALB/c mice	Budesonide or GOS: ↓ eosinophils in BALF GOS + budesonide: **↓** CCL17, CCL22, and IL-33 protein levels; ↓ allergic inflammatory response	—	[60]
Soluble-fiber meal containing probiotic yoghurt; inulin (3.5 g); and the probiotics Lactobacillus acidophilus, Bifidobacterium lactis, and Lactobacillus rhamnosus	Human subjects with stable asthma	Airway inflammation biomarkers, including sputum total cell count, neutrophils, macrophages, lymphocytes, sputum IL-8, and eNO, significantly decreased; no decrease in airway eosinophils	—	[61]
Soluble pectin/insoluble cellulose (4%)	Asthma (female BALB/c mice; OVA induction)	↓ eosinophil inflammation, ↓ frequency of allergic symptoms, ↓ BALF and NALF total cells and eosinophils, ↓ IL-4 in BALF, ↑ IFN-γ and IL-10 in BALF	↑ Bifidobacteria	[62]
*Houttuynia cordata* Thunb. polysaccharides (40, 80, and 160 mg/kg)	Lipopolysaccharide-induced acute lung injury in Balb/c mice	↓ pro-inflammatory cytokine (TNF-α, interleukin-6, and interleukin-1β) production	—	[63]
Citrus pectin-derived acidic oligosaccharides (5%)	*Pseudomonas aeruginosa* lung infection in BALB/c mice	↑ M1 macrophage activation, ↑ IL-10 release, ↓ TNF-α release	↑ *Escherichia coli*, *Allobaculum species*, *Sutturella wadsworthia*, *Bacteroides vulgatus*, *Bifidobacterium* species, *Clostridium difficile*, *Clostridium ramosum*, *Clostridium sphenoides*; ↑ production of butyrate and propionate	[64]
GOS (1%)	HDM-induced asthma in BALB/c mice	↓ AHR development, ↓ BALF eosinophils, ↓ BALF leukocytes, ↓ CCL5 and IL-13	—	[65]
scGOS/lcFOS (in 9:1 ratio)	OVA-induced asthma in female BALB/c and male C57BL/6	↓ OVA-induced AHR; no significant change in the concentrations of IFN-γ, IL-4, IL-5, IL-10, IL-12(p70), IL-13, IL-17, and TNF-α in BAL fluid and plasma	—	[66]
High-fiber diet	HDM-induced asthma in female C57BL/6 and BALB/C mice	↓ total BALF leukocytes, eosinophils, macrophages, and lymphocytes; ↓ IL-4, -5, -13, −10, and IFN-γ; ↓ airway hyperresponsiveness	↑ Bacteroidetes; ↓ Firmicutes	[67]
Apple pectin (30%)	HDM-induced asthma in BALB/c mice	↓ allergic airway inflammation	Changes in intestinal and lung microbiota; increment in SCFAs; ↑ Bacterioidetes; ↑ Bifidobacteriaceae; ↓ Firmicutes	[68]
Chitosan oligosaccharides	OVA-induced asthma in mice	↓ mRNA and protein levels of IL-4, IL-5, IL-13, TNF-α in lung tissue and BALF	—	[69]
FOS (2.5%)	HDM-induced airway inflammation in male C3H/HeN mice	↓ BALF eosinophils, ↓ IL-5	—	[70]
1% *w/w* of 9:1 scGOS: lcFOS; 1% *w/w* of 83% scGOS/lcFOS + 17% pAOS	OVA-induced asthma in male BALB/c mice	↓ OVA-induced airway inflammation and hyperresponsiveness, ↓ BALF inflammatory cells	—	[71]
Raffinose (50 g/kg) and GOS (50 g/kg)	Allergic airway eosinophilia (OVA-sensitized brown Norway rats)	↓ IL-4, ↓ IL-5	↑ total anaerobic bacteria in the colon	[72]
Asian pear pectin (100 µg)	Asthma (OVA-sensitized murine model)	↓ asthmatic reactions in sensitized mice, ↓ IFN-γ, ↓ IL-5	—	[73]
Raffinose (50 g/kg)	Allergic airway eosinophilia (OVA-sensitized brown Norway rats)	↓ IL-4 and IL-5 mRNA expression, ↓ mucus-producing cells, = IFN-γ mRNA expression	↑ bifidobacteria	[74]

Abbreviations: ↑, increased; ↓, decreased; =, no change or no effect; ARDS, acute respiratory distress syndrome; BALF, bronchoalveolar lavage fluid; HDM, house dust mite; TNF-α, tumor necrosis factor alpha; IL-1, interleukin-1; IFN-α, interferon-α; IL-6, interleukin-6; IL-10, interleukin-10; RANTES, regulated on activation, normal-T-cell-expressed and -secreted; IP-10, interferon-inducible protein-10; LPS, lipopolysaccharide; MCP-1, monocyte chemotactic protein-1; MIP-1α, macrophage inflammatory protein-1α; ZO-1, zonula occludens-1, also known as Tight junction protein 1; SD, Sprague–Dawley.

## Data Availability

This study no new data were created.
